# The Occurrence of Etoricoxib-Induced Stevens-Johnson Syndrome With Oral Manifestations in a Female Patient: A Case Study

**DOI:** 10.7759/cureus.63353

**Published:** 2024-06-28

**Authors:** Vasileios Zisis, Petros Papadopoulos, Nikolaos Kyriakou, Christina Charisi, Athanasios Poulopoulos

**Affiliations:** 1 Oral Medicine/Pathology, Aristotle University of Thessaloniki, Thessaloniki, GRC; 2 Hospital Dentistry and Oral Medicine/Pathology, Aristotle University of Thessaloniki, Thessaloniki, GRC; 3 Pediatric Dentistry and Oral Medicine/Pathology, Aristotle University of Thessaloniki, Thessaloniki, GRC

**Keywords:** drug-related side effects and adverse reactions, oral ulcer, oral diseases, stevens-johnson syndrome (sjs), etoricoxib

## Abstract

Stevens-Johnson Syndrome (SJS) constitutes a rather uncommon, and rarely fatal hypersensitivity reaction that primarily impacts the skin and mucous membranes and in certain cases may be attributed to drug administration. The aim of this article is to present a case of etoricoxib-induced SJS in a 46-year-old, female patient. The patient presented herself, as a medical emergency, to the Department of Oral Medicine/Pathology, School of Dentistry, Aristotle University of Thessaloniki, Greece, reporting pain, especially acute pain while eating certain foods, discomfort, dysphagia, and a wound in the left half of the hard palate. The clinical examination revealed a broad ulcer, in the left half of the hard palate as well as multiple ulcerations and erosions in the upper and lower lip. Her medical history was clear; however, the patient mentioned to have received etoricoxib, due to severe back pain, one day prior to our clinical examination. The patient received methylprednisolone 16 mg, twice per day, for two days, followed by methylprednisolone 8 mg, twice per day, for two more days. Her symptoms resigned and since the connection between etoricoxib and SJS was established, the patient was advised to avoid etoricoxib and be wary of adverse effects, when taking drugs especially non-steroidal anti-inflammatory medication. This is one of the first case reports in the literature, linking etoricoxib administration with the emergence of SJS, highlighting the importance of pharmacovigilance. The up-to-date registration of drug-induced adverse effects is of immense importance to protect future patients. SJS does not have a defined treatment strategy. Therefore, most patients are given supportive care and symptomatic treatment, which most commonly involves corticosteroids and antivirals such as acyclovir.

## Introduction

Stevens-Johnson Syndrome (SJS) constitutes a rather uncommon, and rarely fatal hypersensitivity reaction that primarily impacts the skin and mucous membranes [1]. Based on the Gell and Coombs classification of hypersensitivity responses, SJS is categorized as a type-IV (subtype C) hypersensitivity reaction.

The majority of SJS cases may be attributed to the uptake of specific medications [1]. Drugs that may be responsible include antibiotics, anticonvulsants, benzodiazepines, sulfonylureas, diuretics, analgesics, antidepressants, xanthine oxidase inhibitors, androgenic hormones, antineoplastics, immunosuppressants, immunomodulators, corticosteroids, antiparasitic drugs, antiviral drugs, antifungal drugs, antihistamines, acetylsalicylic acid/dipyridamole, and angiotensin-converting enzymes inhibitors as well as angiotensin receptor blockers [2]. Sulfonamides, aminopenicillin, fluoroquinolones, tetracyclines, macrolides, and cephalosporins are loosely associated with the emergence of SJS [2]. Viral infections from herpes simplex virus and Epstein-Barr virus can also lead to the development of SJS [1].

The main factors contributing to the pathological progression of SJS are immunological responses, reactive drug metabolites, and hereditary factors [3]. SJS constitutes a cell-mediated cytotoxic reaction involving CD8+ cells, which results in the death of keratinocytes by apoptosis. The cell-mediated T-cell reactions described are unique to the drug and target the responsible substance. Various drugs that trigger SJS interact with the major histocompatibility complex (MHC) class I and T-cell receptors (TCR), resulting in the proliferation of drug-specific cytotoxic T cells. These cells cause the death of keratinocytes through both direct and indirect mechanisms, which involve the release of cell mediators [4].

Drug-induced reactions are significant and they typically present with non-specific symptoms. Timely diagnosis is crucial, coupled with the identification and prompt discontinuation of potentially harmful medications. It is crucial to prevent further contact with the causative substance. The aim of this article is to present such a case of etoricoxib-induced SJS in a 46-year-old, otherwise healthy, female patient.

## Case presentation

**Figure 1:**
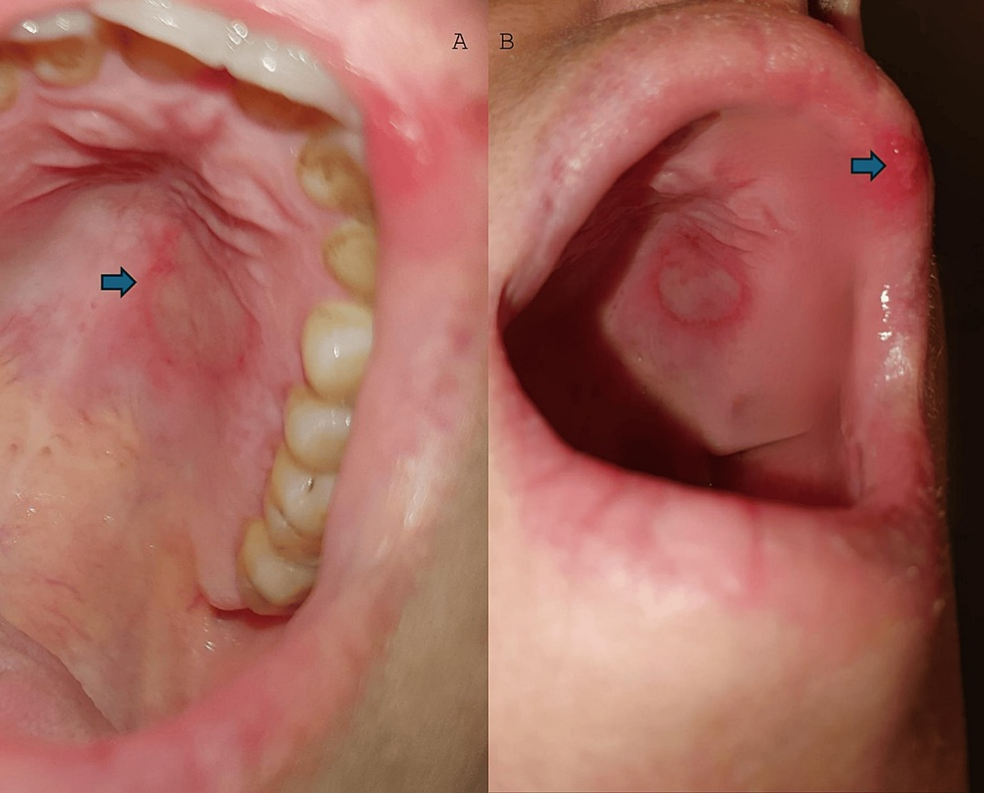
The blue arrows show an ulcer on the hard palate (A) as well as an ulceration on the upper lip (B).

A 46-years-old female patient presented herself, as a medical emergency, to the Department of Oral Medicine/Pathology, School of Dentistry, Aristotle University of Thessaloniki, Greece, reporting constant, severe, and diffuse pain on the maxilla and the lips, especially while eating certain foods, discomfort, dysphagia, and a wound in the left half of the anterior hard palate. The patient signed an informed consent and was, subsequently, examined. The clinical examination revealed a broad ulcer, approximately 1 cm, in the left half of the anterior hard palate, respectively to teeth #14 and 15, as well as multiple ulcerations and erosions in the upper and lower lip. Her medical history was clear; however, the patient mentioned to have received etoricoxib, 60 mg once daily, due to severe back pain, one day prior to our clinical examination (Figure 1).

**Figure 2:**
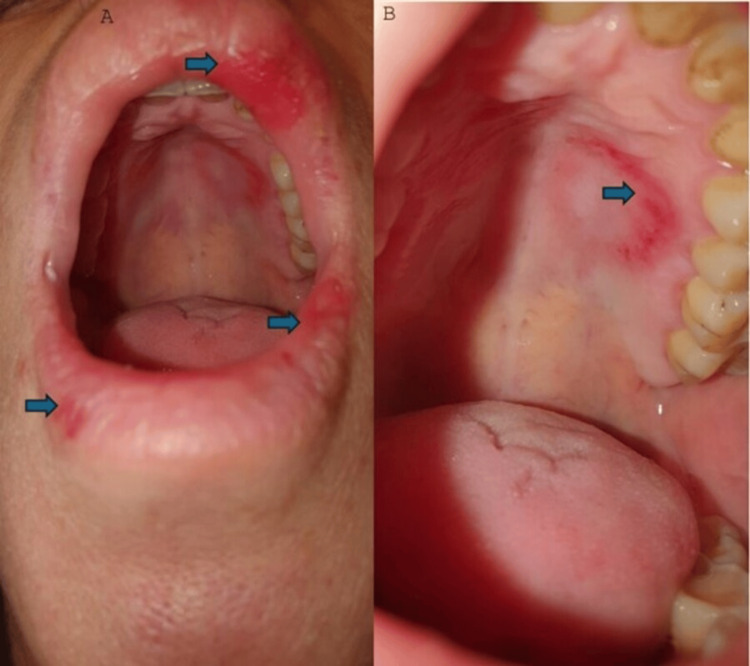
The palatal ulcer and the lip ulcerations have receded, and only widespread erythema remains on the upper and lower lip (A) and on the hard palate (B) (the more distinct areas are distinguished by the blue arrows).

The patient adhered to the following therapeutic regimen: methylprednisolone 16 mgr, twice per day, for two days, followed by methylprednisolone 8 mgr, twice per day, for two more days. At the same time, the patient was requested to apply a topical cream, acyclovir/hydrocortisone, to the intraoral and perioral lesions. On the fourth day of treatment, the patient came for a checkup and was thoroughly re-examined (Figure 2). The patient reported that the pain had started to recede and the wound healing progressed normally.

**Figure 3:**
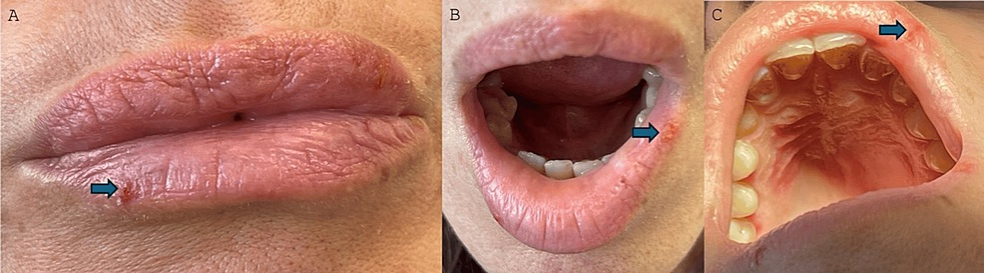
The blue arrows show some minor erosions, which still persist on the lips (A, B, C) whereas the palatal ulcer has completely receded.

The patient discontinued the methylprednisolone and continued to apply the topical cream. The next checkup took place 10 days after the initial examination and the almost complete remission of the lesions was observed (Figure 3).

Since the connection between etoricoxib and SJS was established, the patient was advised to avoid etoricoxib and always be on the lookout for adverse effects, when taking drugs and especially non-steroidal anti-inflammatory medication. The patient took part in regular four-month interval follow-ups without any observed relapse up to date.

## Discussion

Etoricoxib is a medication that specifically inhibits the COX-2 enzyme, which is involved in inflammation. It is mostly used to treat inflammatory conditions such as rheumatoid arthritis, osteoarthritis, and gout. The patent for this medication was granted in 1996, and it received approval for medicinal usage in 2002. Etoricoxib specifically blocks the activity of the cyclooxygenase-2 enzyme. Cyclo-oxygenase plays a role in the transformation of arachidonic acid into prostaglandins (PGs). Prostaglandins significantly contribute to the development of an inflammatory response. Evidence from drug safety studies and limited case reports indicates that etoricoxib is linked to the occurrence of severe cutaneous adverse reactions (SCARs) including SJS, toxic epidermal necrolysis (TEN), and erythema multiforme [5]. In the literature, there are several cases of etoricoxib-induced TEN [3,5,6] and even a fatal one [7]. There is also a case report about etoricoxib-induced SJS [1].

SJS and TEN fall within the same spectrum, with their differentiation lying solely on the percentage of the body surface area that is affected [8]. By definition, SJS affects less than 10%, whereas TEN affects more than 30%. The zone of 10-30% is considered as an SJS/TEN overlap [8]. The incidence of SJS/TEN is expected to range from two to seven cases per million individuals annually [2]. The disease has a gender predilection, with a ratio of females:males 2:1 [9]. HIV and cancer patients manifest more frequently SJS. The prevalence of SJS/TEN in individuals with HIV has been documented to be around 1:1000 patients [10]. The elevated prevalence in HIV patients is attributed to the uptake of several medications, the overall immunological dysregulation, the preexisting genetic polymorphisms, and the underlying, bacterial, viral, and fungal infections of HIV patients. The presence of specific human leukocyte antigens (HLA) has been associated with an elevated risk of developing SJS. Individuals who inherit the HLA-B*15:02 and HLA-B*15:11 are more susceptible to developing SJS when using carbamazepine, particularly among Asian populations [11,12]. HLA-B*58:01 has been associated with allopurinol-induced SJS in both Asian and non-Asian individuals [13]. Additional HLA alleles associated with different medicines include HLA-A*31-01, HLA-A*24:02, and HLA-B*13:01 [14-16]. Polymorphisms in the CYP2C19 gene, which encodes the cytochrome P450 isoform, can also heighten the susceptibility to SJS when exposed to medicines such as phenobarbital, phenytoin, or carbamazepine [17].

SJS typically starts with non-specific symptoms that resemble influenza. These include fever, typically exceeding 38°C, a general feeling of discomfort, pain while swallowing, sensitivity to light, and redness of the conjunctiva. Occasionally, the disease initially presents with skin involvement, characterized by blistering or skin discomfort. The initial phase of SJS is characterized by indistinct erythema, which may be sensitive to touch. The lesions often exhibit bilateral symmetry and do not affect the scalp, palms, or soles of the body. The onset typically occurs in the facial region and subsequently extends to the thoracic area. Frequently, it demonstrates a positive Nikolsky sign. Regular assessment of the total body surface area (TBSA) affected by the disease is crucial as it serves as an indicator of the severity of skin involvement. Mucosal involvement, typically affecting the mouth, eyes, and urogenital area, is frequently observed in approximately 90% of patients [18]. Common ocular manifestations encompass conjunctivitis characterized by the presence of purulent discharge, corneal ulcers, anterior uveitis, panophthalmitis, and trichiasis. The acute phase typically lasts 8 to 12 days. Re-epithelization initiates within a few days and requires a period of two to four weeks to complete [2].

## Conclusions

This is one of the first case reports in the literature, linking etoricoxib administration with the emergence of SJS, highlighting the importance of pharmacovigilance. The up-to-date registration of drug-induced adverse effects is of immense importance to protect future patients. SJS does not have a defined treatment strategy. Therefore, most patients are given supportive care and symptomatic treatment, which most commonly involves corticosteroids and antivirals such as acyclovir. The successful treatment in this case contributes to the understanding of effective management strategies for SJS, even in the absence of a standardized protocol. This case impacts clinical decision-making and patient management, particularly in prescribing NSAIDs and monitoring for adverse reactions.

## References

[1] Kumar PS, Tharuni B (2021). A case report on etoricoxib induced Stevens Johnson syndrome. Indian J Pharm Pract.

[2] Abulatan IT, Ben-David SG, Morales-Colon LA, Beason E, Fakoya AO (2023). A compilation of drug etiologies of Stevens-Johnson syndrome and toxic epidermal necrolysis. Cureus.

[3] Kameshwari JS, Devde R (2015). A case report on toxic epidermal necrolysis with etoricoxib. Indian J Pharmacol.

[4] Ko TM, Chung WH, Wei CY (2011). Shared and restricted T-cell receptor use is crucial for carbamazepine-induced Stevens-Johnson syndrome. J Allergy Clin Immunol.

[5] Kreft B, Wohlrab J, Bramsiepe I, Eismann R, Winkler M, Marsch WC (2010). Etoricoxib-induced toxic epidermal necrolysis: successful treatment with infliximab. J Dermatol.

[6] Moutran R, Maatouk I, Hélou J (2014). Etoricoxib-induced toxic epidermal necrolysis. Int J Dermatol.

[7] Roy SS, Mukherjee S, Era N, Mukherjee M (2018). Etoricoxib-induced toxic epidermal necrolysis: a fatal case report. Indian J Pharmacol.

[8] Roujeau JC (1997). Stevens-Johnson syndrome and toxic epidermal necrolysis are severity variants of the same disease which differs from erythema multiforme. J Dermatol.

[9] Sekula P, Dunant A, Mockenhaupt M (2013). Comprehensive survival analysis of a cohort of patients with Stevens-Johnson syndrome and toxic epidermal necrolysis. J Invest Dermatol.

[10] Mittmann N, Knowles SR, Koo M, Shear NH, Rachlis A, Rourke SB (2012). Incidence of toxic epidermal necrolysis and Stevens-Johnson syndrome in an HIV cohort: an observational, retrospective case series study. Am J Clin Dermatol.

[11] Tangamornsuksan W, Chaiyakunapruk N, Somkrua R, Lohitnavy M, Tassaneeyakul W (2013). Relationship between the HLA-B*1502 allele and carbamazepine-induced Stevens-Johnson syndrome and toxic epidermal necrolysis: a systematic review and meta-analysis. JAMA Dermatol.

[12] Wang Q, Sun S, Xie M, Zhao K, Li X, Zhao Z (2017). Association between the HLA-B alleles and carbamazepine-induced SJS/TEN: a meta-analysis. Epilepsy Res.

[13] Somkrua R, Eickman EE, Saokaew S, Lohitnavy M, Chaiyakunapruk N (2011). Association of HLA-B*5801 allele and allopurinol-induced stevens johnson syndrome and toxic epidermal necrolysis: a systematic review and meta-analysis. BMC Med Genet.

[14] Tempark T, Satapornpong P, Rerknimitr P (2017). Dapsone-induced severe cutaneous adverse drug reactions are strongly linked with HLA-B*13: 01 allele in the Thai population. Pharmacogenet Genomics.

[15] Ozeki T, Mushiroda T, Yowang A (2011). Genome-wide association study identifies HLA-A*3101 allele as a genetic risk factor for carbamazepine-induced cutaneous adverse drug reactions in Japanese population. Hum Mol Genet.

[16] Shi YW, Min FL, Zhou D (2017). HLA-A*24:02 as a common risk factor for antiepileptic drug-induced cutaneous adverse reactions. Neurology.

[17] Manuyakorn W, Siripool K, Kamchaisatian W (2013). Phenobarbital-induced severe cutaneous adverse drug reactions are associated with CYP2C19*2 in Thai children. Pediatr Allergy Immunol.

[18] Letko E, Papaliodis DN, Papaliodis GN, Daoud YJ, Ahmed AR, Foster CS (2005). Stevens-Johnson syndrome and toxic epidermal necrolysis: a review of the literature. Ann Allergy Asthma Immunol.

